# The moderating role of psychosocial work factors in the association between menopausal symptoms and work impairment

**DOI:** 10.5271/sjweh.4306

**Published:** 2026-07-01

**Authors:** Michelle GA Clevis, Sandra H van Oostrom, Bette Loef, MenoPause Consortium, Annegreet Vlug, Annemieke Heijboer, Astrid Bakker, Birit Broekman, Cindy L Kelder, Dorenda van Dijken, Eus van Someren, Eveline Bruinstroop, Fedde Scheele, Fernando Rivadeneira, Irene van Valkengoed, Jacqueline Broerse, Jeanine Roeters van Lennep, John Dierx, Karen Nieuwenhuijsen, Karin I Proper, Maryam Kavousi, WM Monique Verschuren, Peter H Bisschop, Petra Juffer, Richard T Jaspers, Sandra H van Oostrom, Sarah E Siegelaar, H Susan J Picavet, Irene GM van Valkengoed, Karen Nieuwenhuijsen, Karin I Proper

**Affiliations:** 1 Department of Behavior and Health, Center for Prevention, Lifestyle and Health, National Institute for Public Health and the Environment, MA Bilthoven, The Netherlands.; 2 Department of Public and Occupational Health, Amsterdam UMC, Vrije Universiteit Amsterdam, Amsterdam Public Health Research Institute, BT Amsterdam, The Netherlands.; 3 Department of Public and Occupational Health, Amsterdam UMC, Universiteit van Amsterdam, Amsterdam Public Health Research Institute, AZ, Amsterdam, The Netherlands.

**Keywords:** job control, job demand, menopause, social support, work functioning

## Abstract

**Objectives:**

Menopausal symptoms are common and can negatively affect women’s work functioning. Psychosocial work factors may play a role in how women experience these symptoms and their consequences on work. This study aims to examine the association between menopausal symptoms and work impairment, and whether psychosocial work factors moderate this association among peri- and postmenopausal women.

**Methods:**

Data from 9490 peri- and postmenopausal women participating in the Dutch Lifelines cohort were used. Measures included self-reported menopausal symptoms, psychosocial work factors (job demands, work pace, influence at work, job insecurity, social support from colleagues, social support from supervisor, and job control), and work impairment. A two-part modeling approach was applied: logistic regression to estimate the likelihood of reporting any work impairment and a generalized linear model to assess the severity of impairment among those affected. Interaction terms tested whether psychosocial work factors moderated the association between menopausal symptoms and work impairment.

**Results:**

Experiencing more severe menopausal symptoms was associated with both an increased likelihood [odds ratio (OR) 3.04, 95% confidence interval (CI) 2.84–3.26] and greater severity [Exp(β) 1.18, 95% CI 1.15–1.20] of work impairment. No moderating effects were found for most psychosocial work factors. Only job demands moderated the association between menopausal symptoms and the severity of impairment, with the association being stronger among women with high job demands than those with low demands.

**Conclusion:**

Menopausal symptoms contribute to both the presence and severity of work impairment. Although high job demands amplify this association, workplace strategies should support menopausal women across all work conditions.

Menopause, a natural biological transition typically occurring at 45–55 years, is often accompanied by a range of symptoms (1). Prevalent in around 80% of menopausal women, symptoms include vasomotor, psychological, somatic, and sexual symptoms (2). These symptoms vary in both type and severity, with more severe symptoms often having a greater impact on daily functioning, including work performance (3, 4). A growing number of studies report associations between menopausal symptoms and higher levels of absenteeism, reduced work productivity, presenteeism, and an elevated need for recovery after work (5–8). This issue has become increasingly relevant as women’s participation in paid employment has risen across all sectors in recent decades (9). Moreover, demographic shifts such as population ageing and rising retirement ages mean that a growing number of women are working through midlife and the menopausal transition (10).

Psychosocial work factors refer to the characteristics of the work environment that can influence workers’ psychological, physical, and social well-being (11). Emerging evidence indicates that these factors can play a role in shaping the menopausal experience at work. For example, one study reported that all participating middle-aged women identified work-related stress or overload as aggravating their menopausal symptoms (12), which has also been observed in other studies (13, 14). Several psychosocial work factors, including feeling insecure, worrying about one’s job, and lack of appreciation have also been associated with having difficulties coping with menopausal symptoms at work (15). Moreover, multiple studies have reported that support from supervisors and colleagues is associated with lower or less severe menopausal symptom reporting (16–18). Taken together, these findings suggest that psychosocial work factors are linked to the perceived severity of menopausal symptoms, and also individuals’ ability to manage them, and may therefore shape the extent to which such symptoms affect work-related outcomes. Although one study found that menopausal symptoms moderated the relationship between job demands and work ability (19), little is known about whether and how psychosocial work factors themselves moderate the link between menopausal symptoms and work impairment. Understanding how specific work-related factors moderate the relationship between menopausal symptoms and work impairment can help identify groups of women who may be particularly vulnerable to work impairment during menopause. Such knowledge is important for informing workplace policies and interventions that promote women’s health and sustain their participation in the workforce during midlife. Therefore, this study aims to examine the association between menopausal symptoms and work impairment among peri- and postmenopausal women, and whether psychosocial work factors moderate this association.

## Methods

### Study design and population

Data was collected through an additional study implemented in the Lifelines cohort, a multidisciplinary, prospective, population-based study that examines health and health-related behaviors in a unique three-generation design. The cohort includes 167 729 individuals from the north of The Netherlands. The study employed a comprehensive range of investigative methods to assess biomedical, sociodemographic, behavioral, physical, and psychological factors influencing health and disease, with a particular focus on multimorbidity and complex genetics. Recruitment and baseline assessments took place between 2006 and 2013. All participants provided written informed consent. The Lifelines Cohort Study is conducted with the principles of the Declaration of Helsinki and has been approved by the ethics committee of the University Medical Center Groningen, The Netherlands. More details about the study can be found in the cohort profile papers (20, 21).

**Figure 1:**
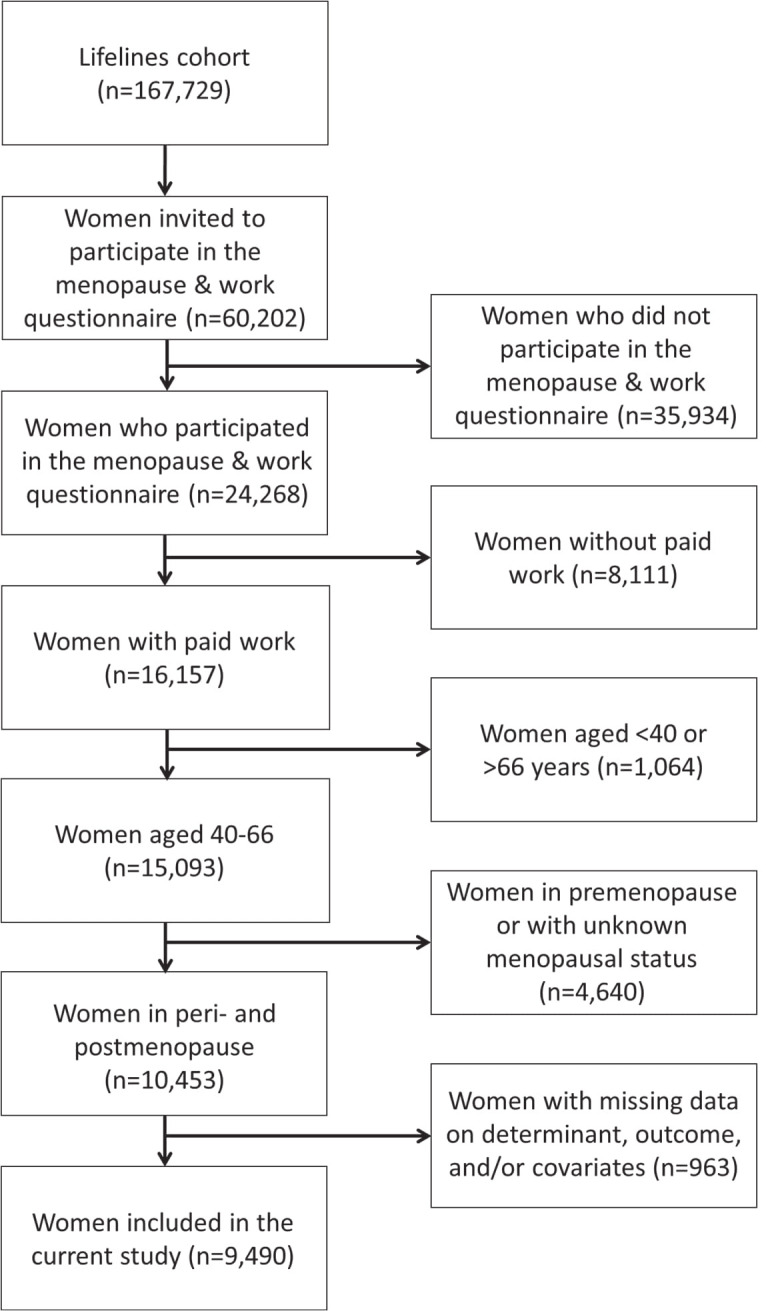
Flowchart of the study population.

In October 2024, all women aged ≥35 included in the Lifelines cohort (N=60 202) were invited to complete an online questionnaire on menopause and work. A total of 24 268 women (40%) participated, of whom 15 093 were aged 40–66 years and in paid work. Of these, 9490 women were in peri- or postmenopause and had fully completed the questionnaire and were therefore included in the current study (figure 1).

### Menopausal status

To assess the menopausal status of the participants, women were first asked whether they were still menstruating. If they responded yes, they were asked whether their menstrual cycle was regular. Those with regular cycles, as well as those who were pregnant or breastfeeding, were classified as premenopausal. Women who were no longer menstruating were asked to indicate the reason. If menopause was the reason they were no longer menstruating, they were asked when their last menstrual period was. Women who experienced irregular cycles for several months or had not menstruated for <1 year were classified as perimenopausal. Those who had not menstruated for >1 year were considered postmenopausal. Participants who had never had a (regular) menstrual cycle, were using hormonal contraception, or had stopped menstruating due to surgery or other medical reasons were categorized as having an unknown menopausal status.

### Menopausal symptoms

Menopausal symptoms were assessed using the Greene Climacteric Scale (GCS) (17), which consists of 21 items. Participants indicated the degree to which they had been bothered by each symptom over the past month. This questionnaire items are divided into four domains; psychological (11 symptoms), somatic (7 symptoms), vasomotor (2 symptoms), and sexual (1 symptom). Responses are scored as follows: 0=not existing, 1=occasionally, 2=often, and 3=very often. The total GCS score range is 0–63 points. A higher score indicates a greater menopausal symptom severity, which reflects a higher number and/or frequency of symptoms.

### Work impairment

The outcome of interest was overall work impairment due to health problems, measured using the Work Productivity and Activity Impairment (WPAI) questionnaire (22). This validated instrument assesses the impact of health on occupational functioning over the past seven days. Overall work impairment was calculated by combining absenteeism and presenteeism.

Absenteeism, representing the proportion of work time missed due to health problems in the past 7 days, was assessed using the following formula:


$$\text{Absenteeism}=\frac{\text{Time missed from work}}{\text{Time missed from work}+\text{Time spent at work}}$$

Presenteeism, reflecting the degree of impairment while at work due to health problems in the past 7 days, was measured with the question: “*During the past seven days, how much did your health problems affect your productivity while you were working?*” Responses were given on a 0–10 Likert scale, where 0=no effect of health problems on work and 10=health problems completely prevented me from working. Scores were divided by 10 to create a fraction between 0 and 1.

Next, work impairment reflects the total percentage of work time missed due to either absenteeism and presenteeism, calculated using the following formula:


Work impairment=(absenteeism+((1−absenteeism)×presenteeism))∗100

The work impairment range is 0–100%, with a higher value reflecting greater work impairment. If either absenteeism or presenteeism was missing, work impairment was coded as missing, except when presenteeism data were absent due to complete (100%) absenteeism. In those cases, work impairment was coded as 100% (23).

### Psychosocial work factors

Most psychosocial work factors were measured using selected items from the Copenhagen Psychosocial Questionnaire (COPSOQ) (24). The following domains were included: quantitative job demands (2 items; eg, “*Do you get behind with your work?*”), work pace (2 items; eg, “*Do you have to work very fast?*”), influence at work (2 items; eg, “*Do you have a large degree of influence on decisions concerning your work?*”), job insecurity (1 item; “*Are you worried about becoming unemployed?*”), social support from colleagues (3 items; eg, “*How often do you get help and support from your colleagues if needed?*”), and social support from supervisors (3 items; eg, “*How often do you get help and support from your immediate superior if needed?*”).

All items were rated on a 5-point Likert scale (always, often, sometimes, seldom, never/hardly ever). Domain scores were calculated by summing the relevant items, resulting in possible ranges of 0–4 for job insecurity, 0–8 for job demands, work pace, and influence at work, and 0–12 for social support from colleagues and supervisors. Higher scores indicated higher levels of the respective domain.

Job control was assessed with two questions from the Dutch Questionnaire on the Experience and Evaluation of Work (VBBA) (25), which included the questions: “*Can you determine the order of your tasks yourself”* and “*Can you organize your work yourself?*”. Both questions were rated on a 4-point Likert scale: always, often, sometimes, and never. The job control range is 0–6, with a higher score reflecting greater job control.

### Covariates

Personal, work-related and lifestyle factors were included as covariates. Age, educational level, and country of birth were obtained from the regular Lifelines questionnaires, with data collection spanning from 2007–2013 (baseline) to 2019–2023 (most recent wave). For each person, the most recent available assessment was used. Occupational group was coded according to the ISCO-08 by Statistics Netherlands. Based on the major ISCO-08 categories, four overarching occupational groups were compiled: high skilled white-collar, low skilled white-collar, high skilled blue-collar, and low skilled blue-collar workers.

All other variables were collected through the supplementary questionnaire on menopause and work, including the following personal factors: household composition (living alone; living with a partner; living with a partner and children; living with children; other), main breadwinner status (ie, being the highest earner in the household; yes/no), provision of informal care (yes/no), and presence of chronic disease (yes/no), and the following work-related factors: working hours, the ability to work from home (not able to work from home; able to work from home but only sometimes or never able to decide when; often or always able to decide when to work from home), and night-shift work (yes/no). Lifestyle factors included alcohol consumption, smoking, and body mass index (BMI). To assess alcohol consumption, participants were asked how many glasses of alcohol they drink on average per week. For smoking, participants were asked whether they were current smokers, former smokers, or had never smoked. BMI was calculated from self-reported weight (kg) divided by height (m) squared.

### Statistical analysis

Descriptive characteristics were reported for the total study population as well as separately by symptom severity groups. Women scoring <18 on the GCS were classified as having no or mild symptoms, whereas those scoring ≥18 (upper tertile) were classified as experiencing severe menopausal symptoms.

To analyze work impairment, a two-part modeling approach was used to account for the highly skewed distribution and large proportion of zero values (meaning no work impairment) in the outcome variable (26). In the first model, we applied logistic regression to estimate the likelihood of having any work impairment (non-zero values), which is further referred to as the presence of work impairment. In the second model, only those participants who reported any impairment (non-zero values) were analyzed. For this group, a generalized linear model (GLM) with a gamma distribution and a log link was used to estimate the associations with the severity of work impairment.

First, we examined the association between the total GCS score and work impairment. Secondly, we examined each psychosocial work factor in relation to work impairment in separate models. To explore potential moderating effects, an interaction term between the GCS score and each psychosocial work factor was included. A P-value <0.10 for the interaction term was used as the threshold to indicate potential moderation. In case of moderation, a stratified analysis was conducted for that specific psychosocial work factor by dichotomizing the factor based on the median value of the scale.

For easier interpretation and practical relevance, the GCS score was divided by a factor of 10, so that the effect estimates reflect the change in the outcome associated with a 10-point increase in the GCS. Models were adjusted for relevant covariates, including personal, work-related, and lifestyle factors. Statistical significance was set at P<0.05 for all associations beside the interaction effects.

All analyses were conducted using R (version 4.4.0).

## Results

### Study population

Table 1 shows the characteristics of the 9490 women included in the current study in total and stratified by menopausal symptom severity. The mean GCS score for the total population was 14.2 [standard deviation (SD) 8.5] and the mean age of participants was 56.8 (SD 5.1) years. Women with more severe menopausal symptoms were, on average, younger (55.4 versus 57.4 years) and less often high educated (41.8% versus 44.5%) than those who experienced no or mild symptoms. They also more often lived with children (40.5% versus 32.2%), were more frequently informal caregivers (33.2% versus 29.1%) and more commonly reported having a chronic disease (55.0% versus 39.4%). Regarding lifestyle, women with severe symptoms were more frequently current (8.0% versus 5.7%) or former smokers (43.5% versus 39.6%) and had a higher average BMI (26.8 versus 25.9 kg/m^2^). Women with severe menopausal symptoms also worked slightly fewer hours per week on average (26.9 compared to 27.4 hours) and were less likely to work night shifts (4.6% versus 5.4%). Also, they were somewhat less likely to be able to decide when to work from home (30.5% versus 34.0%). Across all measured psychosocial work factors, women with more severe symptoms consistently reported more adverse psychosocial conditions than those with no or mild symptoms. For example, women with severe symptoms reported on average lower social support from both supervisors (5.5 versus 5.8) and colleagues (6.3 versus 6.6) and also lower job control (3.4 versus 3.7) (table 1).

**Table 1:** Characteristics of the total study population, stratified on menopausal symptom severity. [GCS=Greene Climacteric Scale; SD=standard deviation]

		Total study population (N=9490)			No or mild symptoms (N=6586)			Severe symptoms (N=2904)^a^	
		Mean (SD)	N (%)		Mean (SD)	N (%)		Mean (SD)	N (%)
GCS score		14.2 (8.5)			9.7 (4.6) *			24.5 (5.9)*	
**Personal factors**									
	Age	56.8 (5.1)			57.4 (5.0) *			55.4 (5.1)*	
Education level									
	Low		1089 (11.5)			735 (11.2)			354 (12.2)
	Middle		4259 (44.9)			2922 (44.4)			1337 (46.0)
	High		4142 (43.6)			2929 (44.5) *			1213 (41.8) *
Country of birth (The Netherlands)			9262 (97.6)			6438 (97.8)			2824 (97.2)
Household composition									
	Alone		1207 (12.7)			854 (13.0)			353 (12.2)
	With partner		4860 (51.2)			3515 (53.4) *			1345 (46.3) *
	With partner and children		2887 (30.4)			1899 (28.8) *			988 (34.0) *
	With children		477 (5.0)			288 (4.4) *			189 (6.5) *
	Other		59 (0.6)			30 (0.5) *			29 (1.0) *
Main breadwinner (yes)			2922 (30.8)			2041 (31.0)			881 (30.3)
Providing informal care (yes)			2878 (30.3)			1915 (29.1) *			963 (33.2) *
Chronic disease (yes)			4189 (44.1)			2593 (39.4) *			1596 (55.0) *
**Lifestyle factors**									
	Alcohol use (glasses/week)	2.6 (5.0)			2.6 (5.0)			2.5 (4.9)	
Smoking									
	Non-smoker		5013 (52.8)			3603 (54.7) *			1410 (48.6) *
	Former smoker		3873 (40.8)			2610 (39.6) *			1263 (43.5) *
	Current smoker		604 (6.4)			373 (5.7) *			231 (8.0) *
Body mass index (kg/m^2^)		26.2 (4.8)			25.9 (4.7) *			26.8 (5.0) *	
**Work-related factors**									
Occupation									
	High-skilled white collar		5473 (57.7)			3826 (58.1)			1647 (56.7)
	Low-skilled white collar		3229 (34.0)			2222 (33.7)			1007 (34.7)
	High-skilled blue collar		201 (2.1)			152 (2.3)			49 (1.7)
	Low-skilled blue collar		587 (6.2)			386 (5.9)			201 (6.9)
Working hours (hours/week)		27.2 (8.9)			27.4 (8.9) *			26.9 (8.8) *	
Job demands (scale 0-8)		3.9 (0.9)			3.9 (0.9) *			4.0 (1.0) *	
Work pace (scale 0-8)		4.3 (1.6)			4.2 (1.6) *			4.6 (1.7) *	
Influence at work (scale 0-8)		4.6 (1.8)			4.7 (1.8) *			4.3 (1.8) *	
Job insecurity (scale 0-4)		0.4 (0.7)			0.3 (0.6) *			0.6 (0.9) *	
Social support from colleagues (scale 0-12)		6.5 (2.4)			6.6 (2.4) *			6.3 (2.4) *	
Social support from supervisors (scale 0-12)		5.7 (2.8)			5.8 (2.8) *			5.5 (2.8) *	
Job control (scale 0-6)		3.6 (1.8)			3.7 (1.8) *			3.4 (1.8) *	
Working from home									
	Able to decide when to work from home		3122 (32.9)			2236 (34.0) *			886 (30.5) *
	Unable to decide when to work from home		1318 (13.9)			877 (13.3) *			441 (15.2) *
	Unable to work from home		5050 (53.2)			3473 (52.7)			1577 (54.3)
Night shift work (yes)			490 (5.2)			357 (5.4) *			133 (4.6) *

a. Women with a GCS score ≥18 (upper tertile) were categorized as experiencing severe menopausal symptoms * P<0.05

### Presence of work impairment

*Menopausal symptoms and presence of work impairment.* In total, 48.2% of the women reported presence of work impairment due to health issues. A higher score on the GCS, indicating more severe menopausal symptoms, was significantly associated with the presence of work impairment. Specifically, each 10-point increase in the GCS score was associated with more than a threefold higher odds of reporting work impairment (OR 3.04, 95% CI 2.84–3.26) (table 2).

**Table 2:** Effect estimates of the associations between menopausal symptoms and the presence of work impairment and interaction terms with psychosocial work factors.^a^ [OR=odds ratio; GCS=Greene Climacteric scale; CI=confidence interval.]

		Presence of work impairment (N=9490)		
		OR	95% CI	P-value
**Main association menopausal symptoms**				
	GCS score (per 10 points)	3.04	2.84–3.26	<0.001
**Main association psychosocial work factors**				
	Job demands (scale 0–8)	1.12	1.06–1.18	<0.001
	Work pace (scale 0–8)	1.03	1.00–1.06	0.07
	Influence at work (scale 0–8)	1.01	0.98–1.05	0.45
	Job insecurity (scale 0–4)	1.23	1.15–1.32	<0.001
	Social support from colleagues (scale 0–12)	1.02	1.00–1.05	0.04
	Social support from supervisors (scale 0–12)	0.98	0.96–1.00	0.02
	Job control (scale 0–6)	0.92	0.89–0.95	<0.001
**GCS score × psychosocial work factors**				
	Job demands (scale 0–8)	1.06	0.98–1.13	0.13
	Work pace (scale 0–8)	1.01	0.97–1.05	0.78
	Influence at work (scale 0–8)	1.01	0.98–1.05	0.48
	Job insecurity (scale 0–4)	0.99	0.91–1.09	0.84
	Social support from colleagues (scale 0–12)	1.00	0.97–1.03	0.99
	Social support from supervisors (scale 0–12)	0.99	0.97–1.01	0.36
	Job control (scale 0–6)	0.99	0.95–1.03	0.58

^a^ Adjusted for age, educational level, country of birth, household composition, being the main breadwinner, providing informal care, having a chronic disease, occupation, working hours, working from home, night shifts, all (other) psychosocial work factors, alcohol use, smoking, and body mass index. Main associations for psychosocial work factors were also adjusted for GCS score.

*Psychosocial work factors and presence of work impairment.* Several psychosocial work factors, except work pace and influence at work, were significantly associated with the presence of work impairment. Higher job demands (OR 1.12, 95% CI 1.06–1.18) and job insecurity (OR 1.23, 95% CI 1.15–1.32) were associated with increased odds of work impairment. In addition, more job control was associated with lower odds of work impairment (OR 0.92, 95% CI 0.89–0.95). Additionally, lower supervisor support (OR 0.98, 95% CI 0.96–1.00) and higher colleague support (OR 1.02, 95% CI 1.00–1.05) were weakly but significantly associated with higher odds of work impairment (table 2).

*Interaction psychosocial work factors and menopausal symptoms.* None of the psychosocial work factors significantly moderated the association between menopausal symptoms and the presence of work impairment. All interaction terms were not statistically significant (P>0.10) (table 2).

### Severity of work impairment

*Menopausal symptoms and severity of work impairment.* Among those reporting any impairment (N=4577), the average work impairment was 42.3% (SD 28.8). A higher menopausal symptoms score was significantly associated with more severe work impairment (table 3). Each ten-point increase in the GCS score was associated with a 1.18-fold increase in the mean work impairment (95% CI 1.15–1.20).

**Table 3:** Effect estimates of the associations between menopausal symptoms and the severity of work impairment and interaction terms with psychosocial work factors.^a^ [GCS=Greene Climacteric scale; CI=confidence interval.]

		Severity of work impairment (N=4577)		
		Exp(β)	95% CI	P–value
**Main association menopausal symptoms**				
	GCS score (per 10 points)	1.18	1.15–1.20	<0.001
**Main association psychosocial work factors**				
	Job demands (scale 0–8)	0.99	0.97–1.02	0.63
	Work pace (scale 0–8)	1.01	1.00–1.03	0.05
	Influence at work (scale 0–8)	0.99	0.97–1.00	0.05
	Job insecurity (scale 0–4)	1.05	1.03–1.08	<0.001
	Social support from colleagues (scale 0–12)	1.00	0.99–1.01	0.78
	Social support from supervisors (scale 0–12)	1.00	1.00–1.01	0.30
	Job control (scale 0–6)	1.01	0.99–1.02	0.37
**GCS score × psychosocial work factors**				
	Job demands (scale 0–8)	1.05	1.03–1.07	<0.001
	Work pace (scale 0–8)	0.99	0.98–1.01	0.48
	Influence at work (scale 0–8)	1.01	1.00–1.02	0.16
	Job insecurity (scale 0–4)	1.02	0.99–1.04	0.21
	Social support from colleagues (scale 0–12)	1.00	0.99–1.01	0.69
	Social support from supervisors (scale 0–12)	1.00	0.99–1.01	0.63
	Job control (scale 0–6)	0.99	0.98–1.01	0.42

^a^ Adjusted for age, educational level, country of birth, household composition, being the main breadwinner, providing informal care, having a chronic disease, occupation, working hours, working from home, night shifts, all (other) psychosocial work factors, alcohol use, smoking, and body mass index. Main associations for psychosocial work factors were also adjusted for GCS score.

*Psychosocial work factors and severity of work impairment.* Regarding psychosocial work factors, higher job insecurity was associated with higher work impairment among those with work impairment [Exp(β)=1.05, 95% CI 1.03–1.08]. Job demands, work pace, influence at work, social support, and autonomy were not significantly associated with the severity of work impairment (table 3).

*Interaction psychosocial work factors and menopausal symptoms.* The interaction analysis showed that job demands significantly moderated the association between menopausal symptoms and the severity of work impairment (P<0.001). No other psychosocial work factor significantly moderated this association (table 3).

Stratified analyses indicated that the association between menopausal symptoms and the severity of work impairment was stronger among participants with high job demands [Exp(β)=1.23, 95% CI 1.18–1.29] compared to those with low job demands [Exp(β)=1.15, 95% CI 1.12–1.19] (table 4).

**Table 4:** Effect estimates of the association between menopausal symptoms and work impairment stratified on median of job demands.^a^ [GCS=Greene Climacteric Scale; 95% CI= 95% confidence interval.]

		Severity of work impairment (N=4577)		
		Exp(β)	95% CI	P-value
Low job demands (N=3345) ^b^				
	GCS score (per 10 points)	1.15	1.12–1.19	<0.001
High job demands (N=1232)				
	GCS score (per 10 points)	1.23	1.18–1.29	<0.001

^a^ Adjusted for age, educational level, country of birth, household composition, being the main breadwinner, providing informal care, having a chronic disease, occupation, working hours, working from home, night shifts, all (other) psychosocial work factors, alcohol use, smoking, and body mass index. Main associations for psychosocial work factors were also adjusted for GCS score. ^b^ Women with job demands score equal or below 4 were categorized as having low job demands.

## Discussion

In this study, we found that higher levels of menopausal symptoms were significantly associated with the presence and severity of work impairment among peri- and postmenopausal women. Of the psychosocial work factors, higher job demands and job insecurity, lower job control, higher social support from colleagues and lower support from supervisor were associated with a higher likelihood of reporting work impairment. Among those with any work impairment, higher job insecurity was associated with greater severity of work impairment. None of the psychosocial work factors moderated the association between menopausal symptoms and the presence of work impairment. However, job demands moderated the association between menopausal symptoms and the severity of work impairment, showing an association that was stronger among women with high job demands compared to those with low demands.

Our findings align with previous studies, supporting that menopausal symptoms are associated with reduced work performance and increased work impairment (6, 7) This again, emphasizes the importance of addressing symptoms to support peri- and postmenopausal women in the workplace. The moderating role of job demands on the association between menopausal symptoms and severity of work impairment suggests that high workload may exacerbate the negative impact of menopausal symptoms. This aligns with prior research indicating that high workload can intensify the experience of menopausal symptoms (12, 13, 17). However, it is important to note that the association between menopausal symptoms and work impairment was strong even among women with lower job demands. This indicates that while job demands may intensify the association, menopausal symptoms on their own represent a substantial risk factor for work impairment. To our knowledge, no previous studies have specifically examined whether job demands moderate the relationship between menopausal symptoms and work impairment. However, one study found that menopausal symptoms moderated the association between job demands and work ability (19). Specifically, they found that the indirect effect of job demands on exhaustion via work ability was significant only among women experiencing high levels of menopausal symptoms. Although the direction of the moderation differs from our study, both findings suggest that there is an interaction between menopausal symptoms and job demands, which can affect work-related outcomes. These results suggest that among women experiencing menopausal symptoms such as sleep disturbances, concentration problems, or fatigue, coping resources may already be limited. Under these conditions, high job demands can place an additional burden that further limits their ability to perform at work (27).

Interestingly, job demands influenced the relationship between menopausal symptoms and the severity, but not the presence, of work impairment. From a methodological perspective, this may be due to the binary measure for presence of work impairment, making it harder to detect subtle moderation effect, compared to the continuous severity measure (28). Consistent with this interpretation, the interaction term for the presence of work impairment showed a similar direction of effect as that for severity but did not reach statistical significance, with a P-value slightly above the predefined threshold of 0.10 for interaction effects.

The lack of moderating effects for other psychosocial work factors indicates that the association between symptoms and impairment was largely similar across different levels of these work factors. Consequently, aside from job demands, there is little indication that other psychosocial work factors define distinct subgroups of women at particularly elevated risk, limiting the need for highly tailored interventions based on these factors. Nevertheless, several psychosocial factors were found to be associated with work impairment itself, including job demands, insecurity, and job control. These findings were in line with previous studies, which also reported that high job demands and insecurity, and low autonomy were associated with lower work productivity among menopausal women (14, 15, 29–31). Improvement of these conditions as part of supportive strategies in work organizations may help reduce work impairment among women during midlife.

### Strengths and limitations

A major strength of this study is the large sample size, which provided sufficient power to detect associations and examine potential moderating effects. Another strength is the availability of detailed information on a wide range of relevant covariates, which allowed for adjustment of potential confounding.

Some limitations should also be noted. First, work impairment was assessed using self-reported data (WPAI), which may have introduced bias. Although the WPAI is a validated and widely used instrument, self-reported measures may not fully correspond with objective measures of work performance, and may over- or underestimate work impairment. Second, menopausal status was based on self-reported menstrual patterns, which may have led to some misclassification. For example, amenorrhea due to low body weight may have been misclassified as menopause. However, only 0.8% of the participants was underweight, and the questionnaire distinguished menopause from other (medical) reasons for absence of menstruation, which likely reduced this risk. Most other self-reported measures used in this study were instruments validated in similar populations, including those for menopausal symptoms and psychosocial work factors, which increases the reliability of the findings.

Furthermore, there is potential selection bias. Comparison of responders and non-responders to the menopause and work questionnaire aged 40–66 years showed that non-responders were, on average, slightly younger, lower educated, and less likely to have The Netherlands as their country of birth (supplementary table S1). This suggests that the study population may not fully represent the underlying population. Moreover, the study population consisted predominantly of highly educated women with a Dutch background, which may limit the generalizability of the results to more diverse populations. In addition, it is also possible that women with more severe menopausal symptoms had already left the workforce, potentially leading to an underestimation of the association between menopausal symptoms and work impairment.

Finally, due to the cross-sectional design, causal conclusions cannot be made about the associations between menopausal symptoms, psychosocial work factors, and work impairment. Future longitudinal studies in more diverse populations are needed to replicate these results and to further clarify the directionality and underlying mechanisms of these associations.

### Concluding remarks

This study showed that menopausal symptoms were associated with both the presence and severity of work impairment among peri- and postmenopausal women. Overall, psychosocial work factors did not consistently moderate this association. Only job demands significantly moderated the association between menopausal symptoms and the severity of work impairment, with higher job demands amplifying the negative impact of menopausal symptoms. While women with high job demands may be more vulnerable to the impact of menopausal symptoms on work impairment, this impact is also significant for women with low job demands. These findings indicate that while reducing job demands may be beneficial, workplace strategies should not be limited to specific subgroups. Instead, broad support for menopausal women in the workplace is warranted, given the overall impact of menopausal symptoms on work functioning.

## Supplementary material

Supplementary material
