# Supplementary material for: The moderating role of psychosocial work factors in the association between menopausal symptoms and work impairment
Source: Scand J Work Environ Health. 2026 Jun 26;52(4):425–33. doi: 10.5271/sjweh.4306 (PMC13349334; doi:10.5271/sjweh.4306)
Supplement: Supplementary material [file SJWEH-52-425-S001.pdf]

The moderating role of psychosocial work factors in the association between menopausal symptoms and work impairment[1]

by Michelle GA Clevis, MSc [2] Sandra H van Oostrom, PhD, Bette Loef, PhD, MenoPause Consortium, Irene GM van Valkengoed, PhD, Karen Nieuwenhuijsen, PhD, Karin I Proper, PhD

1. Supplementary material
2. Correspondence to: Michelle Clevis, Department of Behavior and Health, Center for Prevention, Lifestyle and Health, National Institute for Public Health and the Environment, P.O. Box 1, 3720 BA Bilthoven, The Netherlands. [E-mail: michelle.clevis@rivm.nl]

Supplementary Table S1. Characteristics of responders and non-responders to the Menopause and Work questionnaire aged 40-66

	**Responders to Menopause & Work questionnaire aged 40-66 (n=18,800)**	**Non-responders to Menopause & Work questionnaire aged 40-66 (n=26,300)**
**Age (mean (SD))**	55.5 (6.7)	53.9 (7.3)
**Educational level**		
% low	12.9	18.3
% middle	44.5	46.1
% high	42.6	35.6
**Country of birth (% the Netherlands)**	97.4	96.0

SD, Standard deviation
